# The Status of Food and Nutrition Literacy and its determinants among Elementary School students in Egypt: community nursing-led design

**DOI:** 10.1186/s12912-024-02342-9

**Published:** 2024-10-02

**Authors:** Shaimaa Mohamed Amin, Mutaz DREIDI, Eman Ghallab, Shadia Ramadan Morsy Mohamed, Intima Alrimawi

**Affiliations:** 1 Lecturer of Community Health Nursing, Faculty of Nursing, Damanhour University, Damanhour city, Egypt; 2 Nursing Department, Faculty of Pharmacy, Nursing and Health Professions, Birzeit University, Birzeit, P. O. Box. 14, Palestine; 3 Nursing Education Department, Alexandria University, Alexandris, Egypt; 4 School of Nursing, Georgetown University, 3700 Reservoir Road Northwest, Washington, D.C 20057 USA

**Keywords:** Determinants, Elementary school, Food literacy, Nutrition literacy, Students

## Abstract

**Aim:**

To assess food and nutrition literacy status and its determinants among elementary school students in El-Beheira Governorate, Egypt.

**Background:**

Developing strategies to enhance food and nutrition literacy necessitates a deeper understanding of the food and nutrition literacy situation among elementary school students and associated factors.

**Design:**

A cross-sectional descriptive research design was adopted.

**Methods:**

A final sample of 400 primary school students (aged 6–12 years) at Itay El Barud public elementary schools in El-Beheira Governorate were administered (1) a socio-demographic characteristics and anthropometric measurements questionnaire developed by the researchers, and (2) the Food and Nutrition Literacy Scale. The derived data were analyzed using descriptive and non-parametric tests.

**Results:**

The majority (61%) of students had low food and nutrition literacy scores. The results have shown that overall socio-demographic variables were significant in predicting understanding food and nutrition information, nutritional health knowledge, interactive functional and nutritional literacy, critical food and nutritional literacy, and food labeling.

**Conclusion:**

The study reveals that elementary school students in Egypt have poor knowledge and skills in food and nutrition literacy, largely due to a lack of nutrition education and family awareness. Factors like residential location, income, and education level also contribute to this disparity.

**Implications for the profession:**

To improve nutrition literacy among students, a nursing approach involving community stakeholders and school nurses is recommended. This includes integrating nutrition-related topics into the school curriculum, organizing workshops, and conducting age-appropriate health education sessions. Active engagement between community health and school nurses is crucial for raising awareness about healthy nutritional choices.

**Impact:**

These findings hold an important impact on the education system and those designing curricula, emphasizing the need for significant incorporation of knowledge and skills related to food and nutrition within schools.

**Reporting method:**

Compliance with the STROBE checklist for cross-sectional studies was maintained throughout the research.

**Patient or public contribution:**

No Patient or Public Contribution.

## Background

### Food and nutrition literacy (FNL) factors

FNL refers to a people’s capacity to obtain, comprehend, and apply knowledge related to food and nutrition (F&N), enabling them to make informed decisions about their dietary habits and overall health [1]. It is a crucial component of elementary school education, as well as pediatric health, significantly influencing students’ development, health, and academic performance [1]. Promoting lifelong healthy eating habits at this stage can contribute to reducing the risk of health issues such as hypertension and diabetes in the future [2, 3]. Moreover, assessing the FNL and the factors influencing it among elementary school students is vital in formulating efficient approaches to enhance their understanding of F&N, and to pinpoint areas that require development from school nursing, public health authorities, and other stakeholders [4]. Therefore, it is strongly advised to employ age-appropriate assessment tools to measure their skills and knowledge regularly.

According to the literature, numerous factors can influence FNL, the most fundamental of which is the family environment and acculturation concerning food. Parents and caregivers significantly impact a child’s understanding of F&N by setting an example, which ideally will be one of healthy habits and nutritional guidance [5]. Another influential factor is the school curriculum, as incorporating nutrition education into the curriculum can substantially and directly influence students’ understanding and knowledge of F&N [6–8]. In terms of background predispositions, the socio-economic status (SES) of children’s families significantly shapes their understanding and knowledge of F&N. Children from diverse socio-economic backgrounds may exhibit different access levels to nutritious foods and education; Vazquez& Cubbin ,2020 reviewed the relationship between SES and childhood obesity, revealing a negative correlation in high-income countries and a positive one in low- and middle-income countries (LMICs) [9]. Their review emphasized the importance of implementing interventions to tackle the FNL problem, specifically focusing on children impacted by SES-related concerns. Nevertheless, it is also important to note that the limited access to nutritious foods, especially in LMICs, will physically and chemically impact food security and children’s health, irrespective of their literacy level (i.e., poor people have less access to F&N) [5].

Furthermore, the cultural norms and traditions of children and their families have the potential to impact individuals’ dietary choices and preferences [10]. Understanding the socio-cultural factors that shape food practices is crucial to making informed decisions regarding food policy [10]. Children engage with the food environment they encounter, emphasizing conceptual aspects such as personal identity, gender, religious beliefs, and cultural restrictions [11, 12]. A systematic review examined the cultural factors contributing to childhood obesity in ethnic minority groups and suggested that cultural care practices, such as the consumption of traditional foods and the practice of family meals, can serve as protective factors against childhood obesity [11]. Therefore, it recommends that nurses who work with ethnic minority populations actively promote these practices.

### Prevalence in Egypt

Since 2015, there has been a global increase in the incidence of undernourishment, with the highest prevalence observed in LMICs such as Egypt [13]. In January 2022, Egypt’s population stood at 102.88 million, with 14 million individuals aged between 0 and 4 years [14]. The population distribution in Egypt is predominantly skewed towards the younger age groups [14, 15]. Moreover, the Central Agency for Public Mobilization and Statistics reported that the total enrollment of primary school students in Egypt for the academic year 2020–2021 was 1,337,000 [15]. This indicates the significant number of children in primary schools in Egypt. Nevertheless, although Egypt has made significant progress in increasing school enrollment, approximately 2.1 million children remain unenrolled in formal education [16–18].

Given Egypt’s significant nutrition-related challenges, including high rates of child malnutrition and obesity, the assessment of FNL among primary school students with a view to developing health interventions (e.g., to improve F&N) is becoming crucial [19–22]. UNICEF underscored Egypt’s struggles in achieving Millennium Development Goal 4, which aims to reduce child mortality rates [19]. This is largely due to the persistently high levels of malnutrition and the prevalence of overweight and obesity. Obese or malnourished children are more susceptible to developing non-communicable diseases and deficiencies in essential nutrients [23, 24]. Therefore, understanding and improving FNL can play a key role in addressing these health issues. However, there is a need for more research on this subject in Egypt.

### Aim of the study

This study sets out to explore the FNL status and its determinants among elementary school students in El-Beheira Governorate, Egypt. By doing so, we aim to provide valuable insights that can guide future interventions to improve FNL among this demographic.

### Research questions

- What is the current level of FNL among elementary school students in El-Beheira?
- What are the determinants of FNL among elementary school students in El-Beheira?

## Methods

### Study design

To achieve our research aims, we adopted a cross-sectional descriptive research design, a method known for its ability to provide a snapshot of a specific condition within a population at a given time. This approach, coupled with our adherence to the STROBE checklist for cross-sectional studies, ensures the robustness and reliability of our findings.

### Setting

The study was conducted at Itay El Barud public elementary schools in El-Beheira. This setting was chosen because it has the highest density of primary school students in El-Beheira during the academic year 2022–2023. Itay El Barud educational district has 100 primary schools for boys and girls, located in rural (*n* = 90) and urban areas (*n* = 10). A quarter (*n* = 25) of the schools was chosen randomly, and they had a mean number of enrolled students of 231. All 25 primary schools included in the study are governmental schools. This selection ensured a consistent evaluation of FNL across public educational institutions in the region.

### Participants

The study participants were 400 primary school students (aged 6–11 years). The study’s eligibility criteria included enrollment in primary school and a willingness to participate in the research. The exclusion criteria included any student who was unwilling to participate in the study and had a disability. G Power software (Version 3.1.9.6) was used to calculate the sample size according to the following parameters: total population of 50,000, alpha 0.05, moderate effect size, power of 0.80, and confidence interval of 95%. The calculations revealed that the minimum required sample size was 384, thus 400 participants were recruited to compensate for possible non-response and dropout. We selected 16 students from each school equally by systematic random sampling from each educational grade.

### Measurements

An interview-administered questionnaire was used for data collection, which included two sections, as described below.

#### Socio-demographic characteristics and anthropometric assessment

The researchers developed the socio-demographic section after a thorough review of the literature. It included data such as age, sex, educational grade, place of residence, family income, fathers’ level of education and occupation, and mothers’ level of education and occupation.

The researchers took anthropometric measurements to assess the nutritional status of the students. The researchers used a digital scale to measure the students’ weight while they were wearing minimal clothing and without shoes. The researchers used a wall-fixed tape to measure the students’ height while standing, without shoes, and with their shoulders in a normal position. The body mass index (BMI) was calculated by dividing the body weight by the square of the height. If the body mass index is less than 18.50 kg/m^2^, students are classified as underweight, between 18.50 and 24.99 kg/m^2^ as normal (healthy weight), between 25.0 and 29.99 kg/m^2^ as overweight, and 30.0 kg/m^2^ or higher as obese [25].

BMI-for-age is interpreted using Z-scores to determine weight status. The normal range for BMI-for-age is represented by a Z-score between − 1 standard deviation (SD) and + 1 SD, indicating that the child’s BMI is within the typical distribution for their age and sex. This suggests that the child is maintaining a healthy weight relative to their peers. A BMI-for-age Z-score above + 1 SD is classified as overweight, while a Z-score above + 2 SD indicates obesity. Conversely, a Z-score below − 2 SD is categorized as thinness, and a Z-score below − 3 SD denotes severe thinness [25].

#### Food and Nutrition Literacy Scale

This scale was developed and validated by Doustmohammadian et al., 2017 [26]. The researchers adopted it for the Arabic-speaking target population (as described below), in order to assess the FNL of the studied sample. It included 46 items in the cognitive and skill domains. The cognitive domain was divided into two subscales: “understanding food and nutrition information” (ten items) and “nutritional health knowledge” (five items). The skill domain included four subscales of “functional FNL” (ten items), “interactive FNL” (seven items), “food choice” (six items), and “critical FNL” (four items). Four dichotomous questions assessed food label literacy. The total score of the “Food and Nutrition Literacy Scale” is categorized into three levels: poor (≤ 50%), fair (> 50% to < 74%), and good (≥ 74%).

### Validity and reliability

The study tool was translated into Arabic, the national language and native tongue of students in the studied schools. A jury of five experts in the related field-tested its content validity. The tool’s reliability was tested using Cronbach’s α (*r* = 0.95), indicating reliability. A pilot study was conducted among 40 additional students who were excluded from the final analysis, to ensure the clarity and reliability of the tool.

### Data Collection

Data was collected by the researchers from February to May 2023. The researchers liaised with school administrators to recruit school health nurses for the research. These nurses can serve as key gatekeepers facilitating access for the research team to the studied community, helping to facilitate participant recruitment and data collection. Secondly, the researchers delivered informational sessions or workshops for school nurses about the importance of the research and its potential impact on school students’ nutritional status. These sessions also offered training on research protocols and data collection methods, equipping nurses with the necessary skills to effectively perform their roles in the study.

Each school held meetings with teachers to recruit the participants. After explaining the study’s aims, researchers asked the students if they would agree to participate, and those who did signed the researchers’ written consent form. The researchers then interviewed students in the classrooms and libraries of their own schools from Saturday to Thursday, asking them to complete the questionnaire and obtain their measurements. Each interview lasted about 20 min, and privacy was maintained during the interview and the anthropometric measurements.

### Ethical considerations

Approval was obtained from the Faculty of Nursing Ethics Committee at Damanhour University on August 18th, 2022 (approval code 59-c). Additionally, permission to use the FNL questionnaire was obtained from the author via email. The participants were informed about the aim of the study, and written consent was obtained from them for their participation. Additionally, all parents of the participating children were thoroughly informed about the study objectives, and their written consent was obtained to allow their children’s participation. Confidentiality and anonymity for students were guaranteed by a statement on the cover page. A code number was used instead of a name. Participation was voluntary, and participants were told they could withdraw at any time without consequences.

### Data Analysis

Data analysis was undertaken using SPSS V. 20. The data are presented as the frequencies and percentages for categorical variables. Mean and SD are used to describe core variables. Pearson r correlations are used to assess the correlations between the subscales of FNL. Multiple linear regressions are done to identify the significance of socio-demographic variables in predicting cognitive and skills domains in the FNL Scale. All assumptions were run and checked to meet the regression requirements.

## Results

The study sample consisted of 400 subjects from 25 primary schools, of whom 52% (*n* = 208) were female and 48% (*n* = 192) were male. The average age was 8.6 ± 1.6. In terms of places of residence, more than two-thirds of the sample lived in rural areas (*n* = 276). Approximately 82% of the participants reported that their families had insufficient income to meet their basic needs (*n* = 328). Concerning the parents’ level of education, it was found that 42% of fathers had a university degree (*n* = 168), while 36.5% and 34.3% of mothers had secondary and university education, respectively. About 42% of fathers were in professional jobs (*n* = 169), while 37% of mothers were housewives. According to a standardized Z-score, almost 74% of children were of normal weight (based on height).

The Food and Nutrition Literacy Scale has two subscales: cognitive and skill domains. The cognitive domain is concerned with knowledge relating to understanding F&N information. The skills domain focused on behavior and ways of dealing with food. The data in Table 1 reveals that the participants have a mean of 7.76 ± 3.3 in understanding F&N information and 13.3 ± 3.0 in nutritional health knowledge. These findings indicate that the children have poor cognitive knowledge of FNL. Regarding the skills domain, functional FNL has the highest mean (M = 9.9, SD = ± 5.0), while food label has the lowest (M = 1.9, SD = 2.2). Moreover, Table 2 shows that the sample has poor cognitive and skill abilities regarding FNL. The majority of the sample (61%, *n* = 244) have poor FNL levels, while about a third (32%, *n* = 128) have fair FNL levels, and only 7% (*n* = 28) have good FNL levels.

**Table 1:** Food and nutrition literacy dimensions in children (*N* = 400)

Domain	Mean	SD	Range	Min - Max
Cognitive				
Understanding food and nutrition information	7.76	3.3	32	0–32
Nutritional health knowledge	13.3	3.0	20	0–20
Skills				
Functional food and nutrition literacy	9.90	5.0	30	0–30
Interactive food and nutrition literacy	7.18	4.5	28	0–28
Food choice	5.78	4.2	21	0–21
Critical food and nutritional literacy	3.73	2.4	12	0–12
Food label	1.90	2.2	17	0–17

**Table 2:** Distribution of the students according to their total score of Food and Nutrition literacy (*N* = 400)

Variables	F	%
Poor (≤ 50%)	244	61
Fair (> 50% - < 74%)	128	32
Good (≥ 74%).	28	7

The results demonstrate that there are significant differences in food literacy related to place of residence (*P* = 0.001), family income (*P* = 0.041), fathers’ level of education (*P* < 0.001), mothers’ level of education (*P* < 0.001), fathers’ working status (*P* < 0.001), and mothers’ working status (*P* < 0.001). These findings suggest that children residing in rural areas exhibit lower FNL, as shown in Table 3.

**Table 3:** Distribution of food literacy in relation to socio-demographic characteristics and body mass index (*N* = 400)

Characteristics		Food Literacy						Sig.
		Poor		Fair		Good		
		No.	%	No.	%	No.	%	
Age	6–9	161	40.3	89	22.3	18	4.5	0.749
	10–12	83	20.7	39	9.7	10	2.5	
Gender	Male	123	30.8	56	14.0	13	3.2	0.467
	Female	121	30.2	72	18.0	15	3.8	
Place of Residence	Rural	181	45.3	83	20.7	12	3.0	0.001
	Urban	63	15.8	45	11.2	16	4.0	
Family Income	Enough and save	204	51.0	107	26.8	17	4.3	0.041
	Enough for basic needs only	32	8.0	15	3.7	8	2.0	
	Not enough	8	2.0	6	1.5	3	0.7	
Father’s level of education	Illiterate	33	8.3	6	1.5	1	0.3	0.000
	Primary	28	7.0	5	1.2	3	0.7	
	Secondary	100	25.0	52	13.0	4	1.0	
	University	83	20.8	65	16.2	20	5.0	
Mother’s level of education	Illiterate	58	14.5	7	1.8	1	0.3	0.000
	Primary	34	8.5	13	3.2	4	1.0	
	Secondary	92	23.0	51	12.7	3	0.8	
	University	60	15.0	57	14.2	20	5.0	
Father’s working status	Deceased	2	0.5	3	0.7	0	0.0	0.000
	Farmer	86	21.5	30	7.5	4	1.0	
	Handicraft	35	8.8	11	2.7	1	0.3	
	Not working	0	0.0	0	0.0	1	0.3	
	Professional job	83	20.8	67	16.7	19	4.7	
	Trader	38	9.5	17	4.3	3	0.7	
Mother’s working status	Farmer	31	7.8	14	3.5	3	0.7	0.000
	Housewife	114	28.5	28	7.0	6	1.5	
	Professional job	60	15.0	57	14.2	18	4.5	
	Trader	39	9.7	29	7.3	1	0.3	
Body mass index	Underweight	5	1.3	1	0.3	2	0.5	0.097
	Normal	175	43.7	96	24.0	24	6.0	
	Overweight	39	9.7	16	4.0	0	0.0	
	Obese	25	6.2	15	3.8	2	0.5	

Since all variables are continuous, a Pearson correlation coefficient was run between the F&N subscales. The results show that understanding F&N information has significant positive relationships with nutritional health knowledge (*r* = 0.210, *p* < 0.001), function FNL (*r* = 0.178, *p* < 0.001), food choice (*r* = 0.118, *p* = 0.019), critical FNL (*r* = 0.189, *p* < 0.001), and food label (*r* = 0.841, *p* < 0.001). These results suggest that the more information about F&N, the more knowledge, the more literate, the better the food choice, and the more skills with critical FNL and food label understanding.

Functional FNL was found to be correlated with interactive FNL (*r* = 0.635, *p* < 0.001), food choice (*r* = 0.581, *p* < 0.001), critical FNL (*r* = 0.558, *p* < 0.001), and food labeling (*r* = 0.109, *p* = 0.030). The interactive FNL is highly correlated with food choice (*r* = 0.0.808, *p* < 0.001) and with the critical FNL (*r* = 0.652, *p* < 0.001). Food choice was significantly correlated with critical FNL (*r* = 0.828, *p* < 0.001). All significant relationships were positive in direction, which means that as the first increases, the second will increase, as shown in Table 4.

**Table 4:** A correlation matrix between subscales of food and nutrition literacy dimensions in children (*N* = 400)

Domain	UNFI	NHK	FFNL	IFNL	FC	CFNL	FL
	*r* (*p*)	*r* (*p*)	*r* (*p*)	*r* (*p*)	*r* (*p*)	*r* (*p*)	*r* (*p*)
UNFI		0.210** (< 0.001)	0.178** < 0.001	0.024 0.636	0.118* 0.019	0.189** < 0.001	0.841** < 0.001
NHK	0.210** (< 0.001)		-0.003 0.948	0.051 0.310	0.003 0.955	0.004 0.932	0.012 0.804
FFNL	0.178** < 0.001	-0.003 0.948		0.635** < 0.001	0.581** < 0.001	0.558** < 0.001	0.109* 0.030
IFNL	0.024 0.636	0.051 0.310	0.635** < 0.001		0.808** < 0.001	0.652** < 0.001	-0.042 0.398
FC	0.118* 0.019	0.003 0.955	0.581** < 0.001	0.808** < 0.001		0.828** < 0.001	0.047 0.350
CFNL	0.189** < 0.001	0.004 0.932	0.558** < 0.001	0.652** < 0.001	0.828** < 0.001		0.097 0.052
FL	0.841** < 0.001	0.012 0.804	0.109* 0.030	-0.042 0.398	0.047 0.350	0.097 0.052	

*UNFI* Understanding food and nutrition information, *NHK* Nutritional health knowledge, *FFNL* Functional food and nutrition literacy, *IFNL* Interactive food and nutrition literacy, *FC* Food choice, *CFNL* Critical food and nutritional literacy, *FL* Food label

**correlation is significant at the 0.01 level (2-tailed)

*correlation is significant at the 0.05 level (2-tailed)

A multiple linear regression was done to identify the determinants of food literacy. All assumptions for multiple regressions were met. The results have shown that overall socio-demographic variables were significant in predicting understanding F&N information (*R* = 0.301, R2 = 0.090, F = 4.310, *P* < 0.001), nutritional health knowledge (*R* = 0.240, R2 = 0.058, F = 2.654, *P* = 0.005), interactive functional and nutritional literacy (*R* = 0.224, R2 = 0.050, F = 2.291, *P* < 0.016), critical FNL (*R* = 0.237, R2 = 0.056, F = 2.579, *P* = 0.007), and food label (*R* = 0.280, R2 = 0.079, F = 3.699, *P* < 0.001). Table 5 illustrates a detailed prediction for each studied socio-economic variable; it can be seen that age, gender, place of residence, family income, and mothers’ level of education are significant in predicting the knowledge and skills of FNL.

**Table 5:** Multiple Linear regressions to predict domains of food literacy from demographic and body mass index of study subjects (*N* = 400)

Characteristics	UFNI β (*P*)	NHK β (*P*)	FFNL β (*P*)	IFNL β (*P*)	FC β (*P*)	CFNL β (*P*)	FL β (*P*)
Age	0.717 (0.038)*	0.309 (0.327)	0.205 (0.698)	-0.240 (0.613)	-0.197 (0.657)	0.220 (0.390)	0.197 (0.387)
Gender	0.062 (0.850)	0.066 (0.826)	-0.938 (0.063)	-0.814 (0.071)	-1.011 (0.017)*	-0.435 (0.075)	0.044 (0.837)
Place of Residence	-0.628 (0.102)	0.039 (0.912)	0.818 (0.166)	1.132 (0.032)*	-0.306 (0.535)	-0.705 (0.014)*	-0.402 (0.113)
Family Income	-1.340 (0.003)**	0.668 (0.100)	0.207 (0.761)	0.670 (0.272)	-0.445 (0.435)	-0.410 (0.215)	-0.684 (0.020)*
Father’s level of education	0.368 (0.610)	0.225 (0.733)	-0.402 (0.718)	-0.138 0.890)	-0.170 (0.855)	0.270 (0.616)	0.493 (0.301)
Mother’s level of education	-1.482 (0.011)*	0.050 (0.925)	-1.838 (0.040)*	-1.595 (0.047)*	-1.251 (0.095)	-0.707 (0.103)	-1.129 (0.003)**
Father’s working status	2.221 (0.133)	0.154 (0.909)	-1.576 (0.488)	-0.893 (0.660)	0.451 (0.812)	1.389 (0.207)	1.542 (0.0114)
Mother’s working status	0.958 (0.064)	0.205 (0.665)	-0.262 (0.742)	-1.020 (0.151)	-0.763 (0.251)	-0.234 (0.543)	0.469 (0.169)
Body mass index	1.744 (0.135)	-4.293 (0.000)**	1.929 (0.283)	-2.416 0.133)	-0.949 (0.527)	-0.451 (0.604)	1.411 (0.067)

*UNFI* Understanding food and nutrition information, *NHK* Nutritional health knowledge, *FFNL* Functional food and nutrition literacy, *IFNL* Interactive food and nutrition literacy, *FC* Food choice, *CFNL* Critical food and nutritional literacy, *FL* Food label

**correlation is significant at the 0.01 level (2-tailed)

*correlation is significant at the 0.05 level (2-tailed

## Discussion

### Main outcomes

This paper aimed to assess the status of FNL and its determinants among elementary school students in the El-Beheira. The study, which employed a quantitative cross-sectional approach, sought to fill existing knowledge gaps on this subject in the Egyptian context. The findings from this research add valuable insights to the limited current understanding of FNL in the region. FNL developed during primary education can influence an individual’s health and dietary patterns throughout their lifespan [3]. Therefore, it is crucial to understand elementary school students’ F&N knowledge to create efficient strategies and interventions to improve their FNL and promote healthier lifestyles.

Child malnutrition is a prevalent problem in Egypt; it is primarily caused by limited access to nutritious foods and poor feeding practices, particularly in infants and young children. Recognizing the severity of the problem, UNICEF and the Ministry of Health and Population collaborated to formulate strategies emphasizing early detection and prevention of child malnutrition. These strategies are grounded in evidence-based approaches [16, 19]. The insights derived from this study and other pertinent research contributes to the accumulation of essential evidence required for addressing the issue effectively.

The study findings suggest that over half of the children who participated displayed poor cognitive and skill abilities about FNL. The previous literature suggests that this could be due to the lack of nutrition education in school curricula and low family awareness about this topic [5–7]. Studies conducted in the Middle East revealed low FNL levels in the region, associated with food habits, food-label use, consumption patterns, school performance, food security, dietary diversity, and nutrient adequacy [27–30]. These studies also emphasized the need for more policies and programs to address this issue in this area. Similarly, in Iran, low FNL levels in school-age children were found to be linked to low dietary diversity scores, fruit and dairy diversity, and meat diversity [31]. Such findings highlight the need for improved F&N education.

This study’s findings also indicate a notable disparity in food literacy based on factors such as residential location, household income, fathers’ educational attainment, mothers’ educational attainment, fathers’ employment status, and mothers’ employment status. These findings indicate that children living in rural areas have a lower FNL. Furthermore, factors such as age, gender, place of residence, family income, and mothers’ level of education play a significant role in predicting FNL knowledge and skills. Similarly, numerous previous studies have shown disparities in FNL among children, attributable to variations in urban-rural settings, SES, and regional differences [20, 21]. Therefore, the study results highlight the importance of understanding socio-cultural influences on food practices and suggest the need for diverse government actions to promote sustainable and healthy diets, focusing on identity, gender, religion, and the traditional diets of the Egyptian community.

The study findings revealed that socio-demographic factors considerably influence predicting different aspects of food literacy, such as understanding F&N information, nutritional health knowledge, interactive functional and nutritional literacy, critical FNL, and food label comprehension. Previous studies uncovered the impact of community cultural practices, family dietary habits, and economic constraints on FNL, most specifically highlighting financial hardship’s influence [5, 9, 10]. Therefore, it is crucial that any strategy aimed at increasing FNL among school-age children includes recognition of the component of the influence of family and community in forming children’s eating habits and actively involving parents and caregivers in nutrition education programs. Furthermore, food accessibility and cost issues must be acknowledged and addressed, particularly in lower-income regions. It is also recommended that a more detailed exploration of the socio-demographic factors shaping FNL among Egyptian elementary school students is conducted.

### Recommendations

Various strategies can be implemented to enhance students’ FNL, which necessitate the cooperation of various sectors and professionals within the community, including community organizations, health professionals, local educators, and community leaders. Specifically, the involvement of school nurses is pivotal in leading health education programs. Recommendations include integrating nutrition-related topics into the school curriculum with the Egyptian Ministry of Education. The curriculum should reflect the local culture, incorporating traditional Egyptian cuisine and cooking methods.

Furthermore, workshops led by school nurses can impart nutrition knowledge and skills to primary school students and their families. Age-appropriate health education sessions, particularly focusing on FNL, can be organized with the school nurse leading. The active engagement of community health and school nurses in an ongoing health education program is vital to raising awareness among school-age children about making healthy nutritional choices.

Efficient nurses’ involvement in such programs necessitates a thorough assessment of their current knowledge of F&N, as their influential role can significantly impact students’ dietary habits. Conducting comprehensive surveys and identifying knowledge gaps can guide the development of targeted continuing education programs and professional development workshops. Providing high-quality educational materials, facilitating partnerships with nutrition experts, and offering collaborative learning opportunities can enhance their pedagogical skills and thus the effectiveness of community nursing to improve F&N. Regular evaluations and feedback mechanisms can help monitor the effectiveness of these programs, while advocacy for supportive policies and resource allocation can ensure sustained success over the long term. Such a multifaceted approach can empower school nurses to competently lead nutrition education initiatives, ultimately fostering healthier dietary habits among students.

Furthermore, we recommend providing comprehensive education about F&N for all children through multiple channels, including public health announcements on television, radio, and social media. Emphasizing the benefits of proper nutrition on overall health should be reinforced by influential figures in children’s lives, such as parents, teachers, school nurses, and doctors. To implement this, media platforms should broadcast engaging and informative content, and parents should be encouraged to actively participate in their children’s nutrition education.

Notably, the relatively lower levels of FNL observed among primary school students in Egypt underscore the need for further research. Future studies should focus on interventions to improve FNL in this demographic and any other at-risk student population. To ensure the generalizability of the findings, it is imperative to replicate the study using a larger sample of school-age students from diverse settings. This approach will contribute to a more comprehensive understanding of the factors influencing FNL and facilitate the development of effective interventions for promoting nutritional literacy among primary school students. Engaging in collaborative research with educators, nutritionists, and experts in child development can further improve the quality and applicability of such research.

### Limitations

This study provides valuable insights into the phenomenon of interest; however, it is crucial to recognize its limitations. The study’s limited scope, which was conducted solely in the El-Beheira, restricts the ability to generalize the findings to all children in Egypt. Factors affecting the generalization include cultural variations, socio-economic differences, educational infrastructure, healthcare access disparities, and varied parental involvement between different regions of Egypt. These factors affect FNL across different regions. Despite this limitation, the study included a diverse and comprehensive El-Beheira community sample to enhance representativeness. The final sample included students from various regions, urban and rural areas, and diverse socio-economic backgrounds within the governorate. Therefore, despite the study’s specific relevance to El-Beheira, we included a diverse and representative sample of the local community to enhance our understanding of the topic.

## Conclusion

The study’s findings indicate that the majority of the participating students exhibited low levels of FNL. The regression analysis revealed that age, gender, place of residence, family income, and mothers’ levels of education are significant in predicting FNL knowledge and skills. Therefore, we highly recommend developing efficient strategies and interventions to enhance FNL. These interventions should be tailored to the needs of the Egyptian community.

## Data Availability

The datasets generated during and analyzed during the current study are notpublicly available due to confidentiality agreements but are available uponreasonable request from the corresponding author.
